# Nanofiltration for the Removal of Micropollutants from Surface Water and UV/PAA Oxidation of the Resulting Retentate

**DOI:** 10.3390/ma19112211

**Published:** 2026-05-24

**Authors:** Marta Iwona Bolińska, Janina Piekutin, Urszula Kotowska

**Affiliations:** 1 Department of Technology in Environmental Engineering, Bialystok University of Technology, 45A Wiejska Street, 15-351 Bialystok, Poland; j.piekutin@pb.edu.pl; 2 Department of Analytical and Inorganic Chemistry, University of Bialystok, 1K Ciołkowskiego Street, 15-328 Bialystok, Poland; ukrajew@uwb.edu.pl

**Keywords:** advanced oxidation processes, nanofiltration membranes, peracetic acid, radical degradation kinetics, surface water purification, hybrid water treatment systems

## Abstract

**Highlights:**

- NF allows water purification without prior specialized sample preparation, but is still sensitive to fouling and scaling.
- Many organic pollutants are characterized by strong lipophilicity, which causes them to settle more readily and more intensively on the membrane surface, as well as to dissolve and distribute unevenly in the aqueous environment.
- The UV/PAA system is very effective at removing even concentrated organic contaminants.

**Abstract:**

The occurrence of organic micropollutants (OMPs) in surface waters poses a significant challenge for advanced water treatment systems. At the same time, the management of membrane retentates containing concentrated contaminants remains a critical limitation of membrane-based technologies. In this study, a hybrid treatment approach integrating nanofiltration (NF) with UV/peracetic acid (UV/PAA) oxidation was investigated to address both OMP removal and retentate treatment. NF effectively removed most of the investigated compounds from surface water but generated a retentate with elevated contaminant concentrations. Subsequent oxidation of the NF retentate using the UV/PAA system resulted in rapid degradation of a wide range of micropollutants. Kinetic analysis revealed pseudo-first-order degradation with rate constants ranging from 0.06 to 1.05 min^−1^ depending on compound structure. The highest degradation rates were observed for phenolic compounds, while compounds lacking strongly reactive functional groups exhibited slower oxidation kinetics. Increasing the PAA dose significantly enhanced degradation efficiency and enabled near-complete removal of most contaminants. The obtained rate constants fall within the range reported for radical-based advanced oxidation processes. These results demonstrate that coupling NF with UV/PAA oxidation provides an effective strategy for OMPs removal and treatment of membrane concentrates, supporting the development of integrated technologies for advanced water purification.

## 1. Introduction

The increasing occurrence of organic micropollutants (OMPs) in aquatic environments is one of the most significant challenges in modern water management. These contaminants include pharmaceuticals, personal care products, endocrine disrupting chemicals (EDCs), industrial chemicals, and microplastics, which are increasingly detected in surface waters, groundwater, and treated wastewater worldwide [1,2,3]. Even at trace concentrations ranging from ng/L to µg/L, many of these compounds exhibit high biological activity and may cause chronic toxic effects in aquatic organisms and humans [4,5]. Their persistence, mobility in aquatic environments, and resistance to biodegradation contribute to their long-term accumulation in ecosystems.

Persistent organic pollutants constitute a particularly problematic group of contaminants due to their high chemical stability and resistance to natural degradation. Many OMPs originate from anthropogenic activities and include compounds widely used in agriculture, industry, and consumer products, such as pesticides, plastic additives, pharmaceuticals, and UV filters [1,2]. A key characteristic of these substances is their tendency to bioaccumulate in living organisms and biomagnify along food chains, which may ultimately lead to their presence in the human body [3,4,5]. In addition to their persistence, many of these compounds exhibit endocrine-disrupting properties, interfering with hormonal regulation even at very low concentrations [6,7,8,9]. As a consequence, the presence of OMPs in aquatic environments has attracted increasing attention in both scientific research and environmental policy. Within the European Union, endocrine-disrupting chemicals have become a major topic in water protection strategies due to their potential impacts on ecosystem stability and human health [10,11].

Conventional water and wastewater treatment technologies are often insufficient for the complete removal of these contaminants. Biological treatment processes, which constitute the core of most municipal wastewater treatment plants, are generally designed to remove biodegradable organic matter and nutrients rather than trace organic pollutants. As a result, many micropollutants pass through treatment systems and are continuously released into receiving water bodies. In response to this challenge, advanced water treatment technologies have been increasingly investigated, among them membrane processes, which have gained particular importance.

Membrane technologies comprise a diverse group of separation processes that use semi-permeable barriers to selectively allow certain components to pass while retaining others [12]. These technologies are widely applied in desalination, industrial separations, resource recovery, and water purification [13,14,15]. In recent years, their application in water and wastewater treatment has expanded significantly due to their high separation efficiency and relatively compact process design [16,17]. Among membrane processes, nanofiltration (NF) and reverse osmosis (RO) are particularly effective for removing OMPs from water [18,19,20].

Several physicochemical mechanisms, including steric exclusion, electrostatic interactions, and adsorption at the membrane surface determine the effectiveness of membrane methods in removing OMPs. These mechanisms enable the separation of a wide range of organic compounds with different molecular structures and physicochemical properties [18,19,20]. However, despite their high separation efficiency, membrane processes generate a secondary stream, known as the retentate or concentrate, that contains elevated concentrations of contaminants retained by the membrane [21].

The generation of retentate represents one of the key limitations associated with the large-scale application of membrane technologies in water treatment systems. The chemical composition of retentate depends strongly on the feed water matrix and the nature of the contaminants rejected by the membrane. When membrane processes are used to remove emerging organic pollutants, the resulting retentate may contain a highly concentrated mixture of toxic, environmentally persistent compounds [22,23]. In many practical applications, retentate streams are discharged to municipal wastewater treatment plants or directly into receiving waters, potentially reintroducing concentrated contaminants into the aquatic environment and reducing the overall effectiveness of membrane treatment systems [23]. Therefore, the development of effective and environmentally safe methods for retentate management is essential for the sustainable implementation of membrane technologies.

Among available treatment methods, advanced oxidation processes (AOPs) have been widely investigated for their ability to generate highly reactive radical species that degrade persistent organic contaminants [24]. Traditional oxidation processes used to treat membrane concentrates often rely on Fenton-type reactions involving iron ions. Although these systems can be highly effective, they introduce additional metal ions into the treated medium and may require further post-treatment to remove residual catalysts [24]. Similarly, catalytic oxidation processes using other metal catalysts, including titanium-based systems, may increase operational costs and complicate treatment [25].

In recent years, attention has increasingly focused on oxidation systems based on peracetic acid (PAA). PAA has long been known as an effective disinfectant, but its application in advanced oxidation processes for the degradation of organic micropollutants has only recently been intensively investigated [26,27,28]. When activated by ultraviolet radiation, peracetic acid can generate several highly reactive radical species, including hydroxyl radicals (•OH), acetoxyl radicals (CH_3_C(O)O•), and acetylperoxyl radicals (CH_3_C(O)OO•), which are capable of initiating complex degradation pathways for a wide range of organic contaminants. Previous studies have demonstrated the potential of PAA-based oxidation systems for degrading selected organic micropollutants, particularly bisphenols and pharmaceutical residues [26,27,28].

Despite growing interest in PAA-based oxidation processes, relatively limited attention has been devoted to their application for treating membrane retentates generated during water purification. In particular, studies addressing the treatment of concentrates originating from nanofiltration processes applied to real surface water matrices remain scarce. Understanding the effectiveness of oxidation systems in such complex matrices is essential for developing integrated treatment strategies that address both OMPs removal and membrane concentrate management.

Therefore, the objective of this study was to investigate the effectiveness of an integrated treatment system combining nanofiltration with UV/peracetic acid oxidation for the removal of selected persistent organic pollutants and endocrine disrupting chemicals from surface water. Particular attention was paid to the treatment of the retentate generated during nanofiltration. The study evaluated the removal efficiency of selected OMPs during nanofiltration and examined their degradation in a UV/PAA oxidation system under varying oxidant concentrations and reaction times.

## 2. Materials and Methods

The research was conducted on surface water from the Supraśl River after infiltration, in accordance with ISO 5667 [29], from the raw water tap at the water treatment plant. First, water was allowed to run from the tap for 10 min to remove any contaminants that had accumulated on the tap. Then, samples were collected in clean polypropylene containers with a capacity of 1 L each, after each container was rinsed with raw water. The containers were filled to the brim with raw water, ensuring that no air bubbles remained, which could oxidize the compounds present in the water. The samples were then transported to the laboratory for chemical analysis, as presented in Table 1.

Surface water from the Supraśl River was used for the study, characterized by relatively low color and turbidity (Table 1), which was achieved through filtration in the soil. The relatively high total organic carbon content (40.7 mg/L) is most likely due to the nature of the water: the watercourse is in direct contact with organic matter in the form of aquatic vegetation. It is highly exposed to external factors, such as agricultural activity and subsurface runoff from agricultural fields. Additionally, bottom sediments stirred up by water movement may be a source of TOC in surface water.

The concentrations of ammonium nitrogen and nitrate nitrogen are also relatively low. Comparing them to the results of water tests from the Vistula River conducted by Jakubiak and Bojarski [30], where the nitrate nitrogen content ranged from 3.65 mg/L to 25.64 mg/L, it can be concluded that the Supraśl River is in a significantly better ecological condition than the Vistula River. This is confirmed by the low nitrite nitrogen concentration in the surface water sample (<0.050 mg/L). Nitrite nitrogen is an unstable transitional form between ammonium nitrogen and nitrate nitrogen. This most likely means there was no new inflow of organic pollutants into the Supraśl River, for example, from agricultural runoff or sewage discharge. Furthermore, a high nitrite content in the water would indicate low dissolved oxygen, which in turn would suggest poor ecological status of the watercourse [31].

Physicochemical analysis of surface water samples, organoleptic assessment based on color, turbidity, and suspended solids content, and determination of the SDI_15_ colloidal index for surface water (values ranging from 1.29 to 3.4) made it possible to decide to carry out membrane processes without the need for additional raw water filtration—the decision was supported by the fact that the water had undergone filtration in the ground.

The SDI15 index is determined using a 0.45 µm membrane filter with a 47 mm diameter. The 500 mL of the surface water was filtered with a constant pressure of 2 bars while the time was measured (T_1_). Then, the water was flowing continuously through the installation for 15 min. After this time, it was again measured how many times it would take to filter 500 mL of water (T_2_). The SDI15 index was calculated with the formula:

(1) $${SDI}_{15}=\frac{\left(1-\frac{T_{1}}{T_{2}}\right)*100\%}{15}$$

In the technological research, contaminants from the OMPs group were added to the water being tested. The basic physicochemical properties of the analyzed compounds are presented in Table 2. The concentration of each contaminant in the tested water is equal to 100 µg/L.

Technological research was conducted on a semi-technical pilot plant for the membrane process. The plant was designed by Stadtwerke Düsseldorf (SWD) and implemented by Cornelsen Umwelttechnologie GmbH, Essen, Germany. Grünbeck Wasseraufbereitung GmbH, Höchstädt a.d. Donau, Germany, developed a low-pressure reverse osmosis (LPRO) unit, the GENO-Nano RKF1800 S. Tests were conducted using a spiral module membrane, made of polyamide (PA), with a negative surface charge. The effective filtration area of the membrane was 3.5 m^2^, and the MCWO was 350 Da. A transmembrane pressure of 0.9 MPa was applied. The concentrate flow rate was set at 5 L/h with 30% recovery (conversion), and the permeate flow rate was set at 25 L/h with 70% recovery (conversion). The membrane system operated in cross-flow filtration mode at room temperature for 45 min of continuous operation.

The efficiency of the membrane process was determined by calculating the removal effect, expressed as the retention ratio (R). It was determined using the following formula:

(2) $$R=\left(1-\;\frac{C_{p}}{C_{f}}\right)*100\%$$

where C_p_—concentration in the permeate, C_f_—concentration in the feed (in raw water).

The membrane was preconditioned with distilled water, a sample of surface water containing contaminants was dosed, the nanofiltration process was carried out, and samples of permeate and retentate were collected.

**Figure 1:**
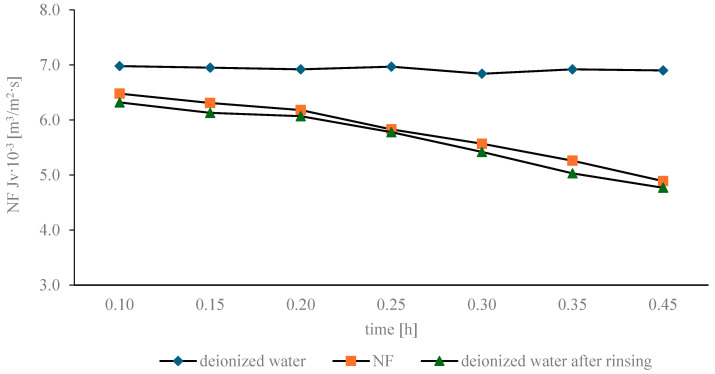
Hydraulic efficiency of the NF membrane.

The efficiency of the filtration process was determined by measuring the permeate volume flux (Jv, m^3^/m^2^·s), the relative water flux α (Jv/Jw), and calculating the retention rate of the concentration of the tested compounds. Hydraulic performance of the NF membrane was presented in Figure 1.

The flux of Jv NF was smaller than the flux of Jv of deionized water at each time of continuous process. This means that the NF membrane was affected by the fouling process, which intensified in time.

The relative water flux α formed from 0.93 to 0.48, which shows the fouling phenomenon. In the first half of the process the Jv NF decrease was stable, but in the second half it sped up, and at the end of the research the Jv NF was 4.89·10^−3^ m^3^/m^2^·s. It showed that the impurities coated the nanofiltration membrane like a thin layer and that this caused the lower hydraulic efficiency of the membrane. The same analysis was prepared for the rinsed membrane, after the actual research. The third Jv values are the lowest obtained, named “deionized water after rinsing”. The difference between the Jv NF and Jv after rinsing ranged between 0.23 and 5·10^−3^ m^3^/m^2^·s, depending on time, which means the fouling and the membrane clogging was not permanent and that the NF membrane could be reused.

The obtained retentates were adjusted to pH 9 by adding 1 M NaOH and then subjected to oxidation in a UV/PAA system. The pH value of 9 was selected based on previous UV/PAA studies on structurally related micropollutants, which showed that alkaline conditions enhanced PAA activation and degradation efficiency [32,33]. The oxidation experiments were performed using the same laboratory photochemical setup and operating mode as previously applied for PAA-based oxidation of structurally related micropollutants [32,33]. Briefly, 30 mL of NF retentate was placed in a 100 mL reaction vessel and mixed with the appropriate amount of PAA. Two PAA concentrations were used: 1·10^−3^ mol/L and 5·10^−3^ mol/L. The samples were exposed to UV-C radiation at 254 nm using a Herolab UV lamp (GmbH Laborgeräte, Höchstädt, Germany) with a nominal power of 15 W. The reaction mixture was continuously stirred at 200 rpm to ensure homogeneous irradiation and uniform distribution of the oxidant. The experiments were conducted in an air-conditioned laboratory, with the ambient temperature maintained at 21 °C throughout. The oxidation time ranged from 1 to 90 min. At selected reaction times, the radical reaction was stopped by adding 720 µL of 20% Na_2_S_2_O_3_ solution. The concentrations of the investigated OMPs were then determined in the post-reaction mixtures.

Analytical determinations and gas chromatography with mass spectrometry (GC–MS) were performed in accordance with applicable standards or using analytical methodologies commonly recognized and recommended in the scientific literature. The test results presented in the paper are the average of at least three simultaneous determinations.

The tested compounds were isolated from water samples using the ultrasonic-assisted microextraction (USAME) technique.

For this purpose, the water sample was placed in a 25 mL volumetric flask with a previously weighed portion of 0.75 g disodium hydrogen phosphate (4%). Next, 5 mL of the obtained solution was placed in four conical test tubes, to which 100 µL of chlorobenzene (extraction solvent) and 250 µL of acetic anhydride (derivatization reagent) were added. Extractions were carried out in an ultrasonic bath at 42 kHz and 230 W for 5 min at room temperature, until an emulsion was obtained. To separate the phases, the obtained solutions were centrifuged at 4000 rpm for 5 min in a laboratory centrifuge.

The organic phase which accumulated at the bottom of the conical tubes was collected using a 100 µL Hamilton syringe (Reno, NV, USA) and transferred to a 150 µL microvial with an integrated insert. The organic-phase samples prepared in this way were analyzed by GC–MS.

The extracted samples were analyzed by gas chromatography–mass spectrometry (GC–MS). For this purpose, an HP 7890B gas chromatograph (Agilent Technologies, Santa Clara, CA, USA) with an electronic pressure control device was used. The analyzer was connected to an MSD 5977A mass detector (Agilent Technologies, Santa Clara, CA, USA), using a 30-m HP-5MS column (5% phenylmethylsiloxane) with an internal diameter of 0.25 mm. Injection was performed in splitless mode at 250 °C. The device used helium as a carrier gas with a constant flow rate of 1.0 mL/min and a purity of 99.999%. The electron impact source temperature was 230 °C with an electron energy of 70 eV. The quadrupole temperature was 150 °C, and the temperature of the interface between GC and MS was 280 °C. To interpret the results of the chromatographic analysis, after data integration, two calibration curves were prepared for each compound—one curve for low concentrations of pollutants, by plotting points with known concentrations of 1 µg/L, 5 µg/L, 10 µg/L, and 50 µg/L, and a second curve for high concentrations of pollutants, by plotting points with known concentrations of 50 µg/L, 100 µg/L, 200 µg/L, 300 µg/L, and 500 µg/L. Equations were determined for each of the curves, based on which the results of the chromatographic analysis were converted to obtain the results of pollutant concentrations in the prepared samples. The obtained calibration curves showed linearity in all concentration variants and throughout the entire analyzed concentration range. The values of the determination coefficients (R^2^) for the obtained equations ranged from 0.984 (E2) to 0.999 (DEET, BPh, and 4MBC).

## 3. Results and Discussion

### 3.1. Membrane Process

The removal efficiency of the tested organic pollutants by nanofiltration varies considerably and is presented in Table 3.

The best removal efficiency was achieved for CTM, equal to 99.5%. Similar removal effects, exceeding 98%, were observed for TRC (98.9%), 4MBC (98.5%), and E2 (98.2%). Significantly weaker removal effects were observed for DEET (62.3%) and 4-OP (21.2%). The effectiveness of 4-OP removal is a matter of debate. Research conducted by D. Mroczko et al. [34] indicates that nanofiltration removed all analyzed contaminants, including 4-OP. However, the fundamental difference was that the study by Mroczko et al. [34] used a mixture of smaller amounts of organic substances, resulting in fewer interacting factors.

Furthermore, although the study was conducted on surface water from the Mała Panew River, the water underwent preparatory processes, i.e., coagulation and sedimentation, which could have significantly reduced the content of interfering substances. However, Kappel’s analysis [35] emphasizes that many organic substances, including 4-OP, are difficult to remove using membranes, with efficiency often falling below 70%. For this reason, the use of additional membrane process stages should be considered [35]. This suggests that further studies should be conducted to accurately observe 4-OPs behavior on membranes.

The removal efficiency of TRC, at almost 99%, is satisfactory, especially given that its retention coefficient is typically 38–67% (depending on the membrane subtype). When NF is combined with biological methods, it achieves approximately 84% efficiency, according to research by Wendt et al. [36]. However, it should be noted that this research was conducted on a different medium—wastewater that had been previously treated with traditional biological methods before the membrane process.

DEET proved difficult to remove, despite its relatively high concentration in raw water. This was in contrast to the studies by Acero et al. [37], in which NF membrane removal efficiency ranged from 92.1% to 100%, depending on the water matrix. The studies by Acero et al. were based on surface water and treated wastewater. They were distinguished by lower chemical oxygen demand and lower organic carbon content than water from the Supraśl River. The problematic nature of DEET was also demonstrated in an earlier series of studies [38]. This means that research on DEET should continue to explore the mechanism of its removal from water using membrane methods.

The low concentration of 4-OP in the feed reflects the prevailing conditions in the aquatic environment. Studies by Salvaterra et al. [39] showed that concentrations of 4-OP in real conditions in watercourses are even lower, approaching the detection limit. In two of the three samples tested, 4-OP concentrations of 0.027 µg/L and 0.008 µg/L were detected, which are more than 20 times lower than those obtained under laboratory conditions. All compounds analyzed in this study have a log K_ow_ value greater than 2 (Table 2) [40]. They are characterized by moderate to strong lipophilicity [41], so they settle readily and intensively on the membrane surface, as clearly evident in the obtained results.

Analyses conducted by Yaqub et al. [42] indicate that cations present in water, mainly magnesium and calcium, cause scaling, i.e., clogging of membrane pores by the deposition of ions forming a sediment similar to limescale. Osmotic membranes are particularly sensitive to this scaling due to their very small pore size, typically ranging from 0.0001 µm to 0.001 µm, compared with nanofiltration membranes, but nanofiltration membranes can still be affected by this phenomenon.

In addition, scaling is accompanied by fouling, i.e., the deposition of remaining contaminants on the membrane. Microorganisms present in water can settle here, forming biofilm and suspended particles [43]. Its direct effects include disturbances in the flow of feed through the membrane, difficult-to-control pressure surges in the system, difficulties in rejecting contaminants by the membrane, and ultimately damage to the osmotic membrane, resulting in a significant deterioration in permeate quality [43,44,45,46,47].

**Figure 2:**
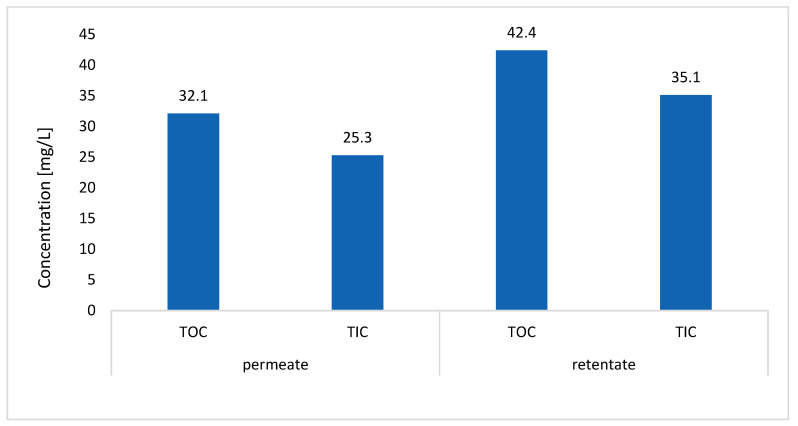
Results of total organic carbon and total inorganic carbon tests.

The TIC and TOC results, measured with Hach LCK380 TOC cuvette tests, in the concentrate after NF are slightly higher than in raw water (Figure 2, Table 1), but the permeate tests show decreases in TIC and TOC values. This means that fouling has occurred, resulting in carbon compounds being retained on the membrane surface.

### 3.2. Oxidation Efficiency of the Concentrate in the UV/PAA System

**Figure 3:**
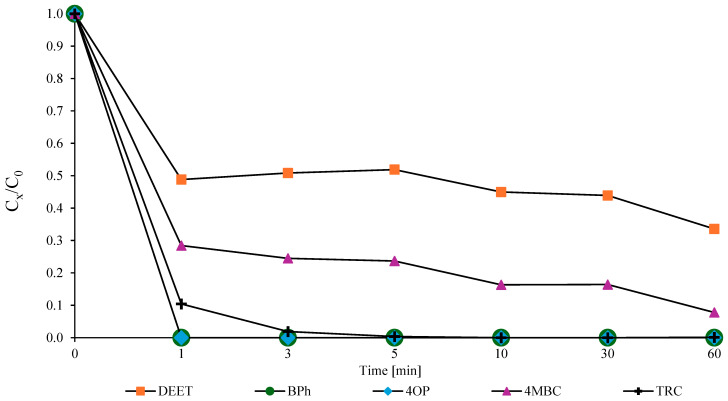
The effect of NF concentrate oxidation with 1·10^−3^ mol/L PAA, depending on time elapsed—part 1.

**Figure 4:**
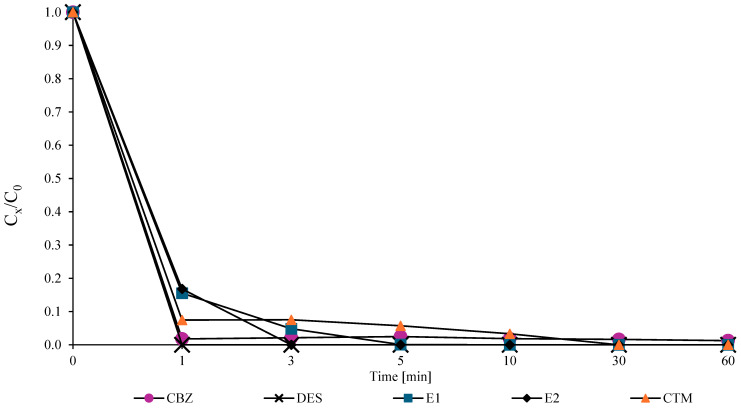
The effect of NF concentrate oxidation with 1·10^−3^ mol/L PAA, depending on time elapsed—part 2.

**Figure 5:**
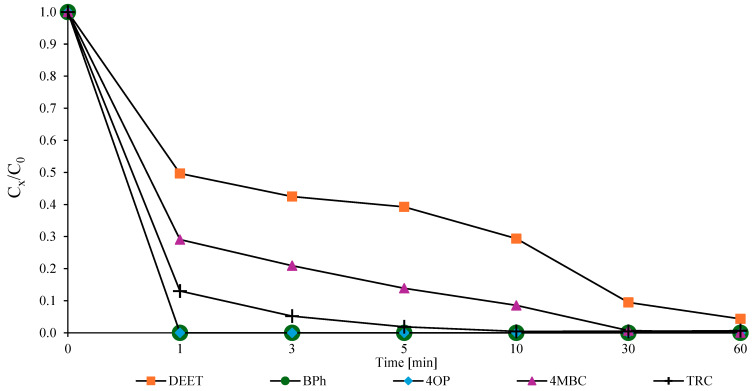
The effect of NF concentrate oxidation with 5·10^−3^ mol/L PAA, depending on time elapsed—part 1.

**Figure 6:**
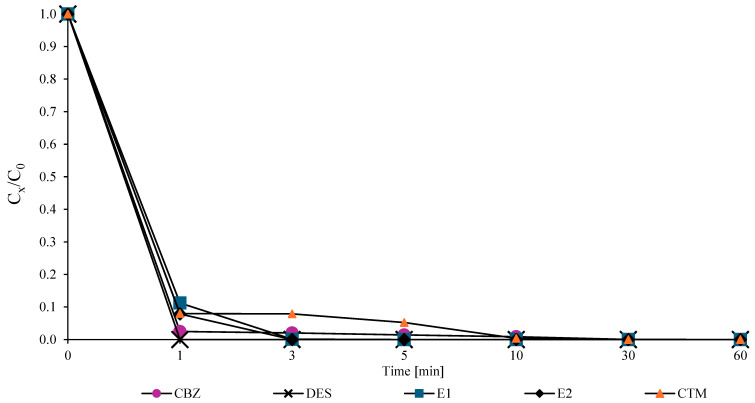
The effect of NF concentrate oxidation with 5·10^−3^ mol/L PAA, depending on time elapsed—part 2.

The following oxidation times were adopted for the nanofiltration concentrate: 1 min, 3 min, 5 min, 10 min, 30 min, and 60 min. Two series of tests were performed at two oxidant concentrations: 1·10^−3^ mol/L (Figure 3 and Figure 4) and 5·10^−3^ mol/L (Figure 5 and Figure 6). To determine the effect of contaminant removal, the C_x_/C_0_ ratio was calculated, where Cx is the concentration of the contaminants after a given oxidation time and C_0_ is the base concentration of the contaminants in the concentrate.

The results of NF concentrate oxidation with 1·10^−3^ mol/L PAA showed that, for all contaminants, the majority of removal occurred in the first minute. During this time, it was possible to eliminate 90% or more of BPh, 4-OP, DES, TRC, and CTM. The weakest reaction was observed with DEET, which was reduced by approximately 50%, and 4MBC, of which 28% remained.

Reviews of the literature emphasize 4MBC’s resistance to removal by light-based methods. This is because 4MBC exhibits a certain resistance to solar radiation. In the UV/PAA system, UV radiation is intended to generate free radicals from PAA, but it also plays a major role in removing organic contaminants. Here, it is clear that only the oxidizer itself, peracetic acid, affected 4MBC, as evidenced by the observed residue of the substance [48,49]. This means that 4MBC is a compound that is not very susceptible to oxidation, which is also due to its molecular structure.

In the third minute of oxidation, most of the remaining contaminants were eliminated. Slightly more time was required to oxidize CTM, whose concentration in the concentrate gradually decreased significantly until the 30th minute, when it was completely removed. The oxidation method used by Li et al. [50] also proved effective in removing TRC. Within the first few minutes of the photo-Fenton process oxidation, 90% of TRC was removed. In the following minutes, this effect increased very slowly, reaching nearly 100% effectiveness after 30 min [50].

This effect is slower than that shown in the results, as the UV/PAA system achieved an efficiency of over 98% after just 3 min. DEET proved to be the most resistant compound, with irregular concentrations during oxidation, most likely due to the influx of light during the reaction arrest in the other oxidized concentrate samples. However, this does not change the fact that after 60 min of oxidation, only 66% of it was removed. This is another example of the problems associated with this compound. Although it is widely used as a popular repellent, it poses a challenge to remove from the aquatic environment in the least invasive manner possible.

The literature data confirms that a UV-activated PAA is an effective method for removing organic contaminants, including pharmaceutical residues [32,33]. They also confirm that the compounds with the lowest molar mass underwent the slowest oxidation: DEET (191.27 g/mol), BPh (182.22 g/mol), and 4-OP (206.32 g/mol).

A fivefold increase in the oxidant dose improved the oxidation of the concentrate after NF (Figure 5 and Figure 6).

In the first minute of oxidation, very similar results were obtained for the removal of most contaminants, except for E1 and E2. E1 was removed in an amount 5% greater than with 1·10^−3^ mol/L PAA, and the oxidation effect for estradiol is even more pronounced—with 1·10^−3^ mol/L PAA, after 1 min of oxidation, approximately 17% remained relative to the baseline concentration, while with 5·10^−3^ mol/L PAA, only 11% remained.

For other contaminants, a clear effect of increasing the oxidant dose is also observed at subsequent oxidation times. At 3 min, E2 was completely removed, while E1 remained at trace levels. After 60 min of oxidation, DEET was removed by 96%, while 4MBC was completely removed.

This analysis of the effectiveness of organic pollutant oxidation has demonstrated that the UV/PAA oxidation system can remove most pollutants within 60 min. The fastest reduction of pollutants occurs within the first minute of oxidation. The use of 1·10^−3^ mol/L PAA is efficient for most compounds, except DEET, 4MBC, and 4-OP, which require a fivefold higher oxidant concentration for efficient degradation.

### 3.3. Kinetics of UV/PAA Degradation of OMPs in Nanofiltration Retentate

The degradation kinetics of micropollutants during oxidation of the nanofiltration concentrate in the UV/PAA system were evaluated using linearized kinetic models. Before selecting the final kinetic model, the concentration–time data were graphically analyzed using both pseudo-first-order and second-order relationships. The pseudo-first-order model was assessed by plotting ln(C/C_0_) against reaction time, whereas the second-order model was evaluated by plotting 1/C against reaction time according to 1/C = 1/C_0_ + k_2_t. The ln(C/C_0_) versus time plots showed better linearity, indicating that the degradation of the investigated OMPs was more accurately described by pseudo-first-order kinetics. This result is also consistent with the experimental conditions, in which OMPs were present at trace concentrations. In contrast, PAA and UV-generated reactive oxidizing species were present in excess relative to the target compounds. Under such conditions, the concentration of oxidizing species can be treated as approximately constant over the analyzed degradation period, and the apparent degradation rate can be expressed primarily as a function of OMP concentration. Therefore, the pseudo-first-order model was used to determine the apparent degradation rate constants according to the following equation:

ln(C/C_0_) = −kt (3)

where C_0_ is the initial concentration of the compound, C is the concentration at time t, and k is the apparent pseudo-first-order rate constant.

The half-life time, t_1_/_2_, was calculated from the pseudo-first-order rate constant according to the equation t_1_/_2_ = ln2/k. This parameter was used as an additional indicator of the susceptibility of individual OMPs to oxidation in the UV/PAA system; lower t_1_/_2_ values indicate faster degradation and higher oxidation susceptibility. The pseudo-first-order rate constants and calculated half-life times obtained in this study are summarized in Table 4.

The literature data indicate that degradation rate constants for organic micropollutants in radical-based advanced oxidation processes typically fall within the range of approximately 0.01–1.5 min^−1^, depending on compound structure, oxidant system, and water matrix [27,28,33,50,51,52]. The literature data on pseudo-first-order kinetic oxidation k-values for analyzed OMPs are given in Table 5.

The obtained results show that oxidation rates vary significantly with the chemical structure of the investigated compounds. Phenolic EDCs such as 4-OP, E1, and E2 exhibited the fastest degradation kinetics (k > 0.6 min^−1^). The high reactivity of these compounds can be attributed to the presence of phenolic functional groups, which readily react with hydroxyl radicals generated during advanced oxidation processes. In contrast, compounds lacking strongly reactive functional groups, such as DEET, showed significantly lower degradation rates. To evaluate the efficiency of the UV/PAA system, the obtained kinetic constants were compared with values reported for other advanced oxidation processes used for micropollutant degradation. The comparison shows that the rate constants obtained in the present study fall within the range reported for UV-activated peracetic acid systems and other radical-based AOPs. Previous studies have demonstrated that degradation rates of OMPs in UV/PAA systems typically range from approximately 0.05 to 1.2 min^−1^, depending on the compound structure and reaction conditions [26,27,28].

The degradation behavior of the investigated OMPs in the UV/PAA system can be explained by the formation and subsequent reactions of radical species generated during the photolysis of peracetic acid. Under UV irradiation, PAA undergoes homolytic cleavage, leading to the formation of highly reactive radicals, primarily hydroxyl radicals (•OH) and acetoxy radicals (CH_3_COO•):

CH_3_COOOH + hν → CH_3_COO• + •OH (4)

The acetoxy radical may subsequently react with dissolved oxygen to form acetylperoxyl radicals (CH_3_COOO•), which also participate in oxidation reactions of organic compounds. These radicals exhibit high oxidation potentials and can react with organic molecules via mechanisms such as hydrogen abstraction, electron transfer, and aromatic ring addition. Previous studies indicate that HO• is an important oxidizing species in UV/PAA systems, although PAA-derived organic radicals may also contribute substantially depending on the target compound and water matrix [26,27,28,53,54]. The degradation kinetics observed in this study indicate that their molecular structure strongly influences the reactivity of the investigated OMPs. Phenolic endocrine-disrupting compounds such as 4-OP, E1, and E2 exhibited the fastest degradation rates. This behavior can be attributed to the presence of phenolic functional groups that readily undergo electrophilic attack by hydroxyl radicals. Such reactions typically proceed through radical addition to the aromatic ring, forming hydroxylated intermediates that subsequently undergo ring-opening reactions and further oxidation [32,33]. Aromatic compounds containing electron-rich functional groups, such as BPh and DES, also showed relatively high degradation rates. Their susceptibility to radical oxidation is associated with electron transfer reactions and the formation of hydroxycyclohexadienyl radicals as intermediate species. Similar degradation pathways for aromatic micropollutants have been reported in studies investigating UV-based advanced oxidation processes [26,27,28]. In contrast, compounds with more stable molecular structures exhibited slower degradation kinetics. The UV 4MBC showed moderate degradation rates in the UV/PAA system. UV filters are designed to absorb ultraviolet radiation and resist photochemical degradation, which explains their relatively high stability under oxidative conditions. Similar behavior has been observed in other AOPs, where UV stabilizers degrade more slowly than phenolic endocrine disruptors. CBZ also showed moderate degradation kinetics. Although it is known to persist in conventional biological wastewater treatment processes, several studies have demonstrated that AOPs can efficiently degrade it. Radical attack on aromatic and heterocyclic rings leads to the formation of hydroxylated intermediates, which are then further oxidized and fragmented [50]. Among the investigated compounds, DEET showed the lowest degradation rate. The relatively high resistance of DEET to advanced oxidation processes has also been reported in previous studies. It is associated with the absence of strongly electron-donating functional groups in its molecular structure. Consequently, degradation occurs mainly through slower hydrogen abstraction reactions and multi-step oxidation pathways.

The compound-dependent degradation kinetics observed in this study may also provide indirect information on the relative contribution of different reactive species in the UV/PAA system. Although radical-quenching experiments were not performed, the obtained trends are consistent with published mechanistic studies on UV/PAA and Fe/PAA processes [26,53,54,55,56,57]. The rapid degradation of phenolic and electron-rich compounds, particularly 4-OP, E1, E2, and DES, suggests a major contribution from highly reactive oxidants, such as HO•. In contrast, the slower degradation of DEET, CBZ, and 4MBC indicates that their transformation was probably controlled by less direct, multi-step oxidation pathways, in which PAA-derived organic radicals may also contribute. This interpretation is particularly relevant for the NF retentate matrix. In the presence of natural organic matter and inorganic radical scavengers, HO• can be rapidly consumed by background constituents. In contrast, more selective PAA-derived organic radicals may retain greater relevance for OMP transformation [53,54,55]. Therefore, the degradation observed in this study should be interpreted as a combined radical process rather than as the effect of a single dominant oxidant. In addition, the measurable iron content in the investigated surface water may have promoted partial Fe-assisted PAA activation, which has been reported to involve carbon-centered radicals and/or high-valent iron species [26,56,57]. Quantitative differentiation of the contribution of HO•, CH_3_C(O)O•, and CH_3_C(O)OO• would require dedicated quenching experiments or probe-compound kinetic modeling and should be addressed in future mechanistic studies.

The high removal efficiencies obtained in this study strongly support the applicability of UV/PAA oxidation as an effective treatment step for reducing the micropollutant burden in NF retentate. In addition to the rapid disappearance of parent OMPs, the reaction mechanisms reported for PAA-based AOPs indicate progressive structural transformation of contaminants, often involving cleavage or modification of functional groups responsible for persistence, hydrophobicity, or biological activity [32,33,53,54,58]. Therefore, UV/PAA treatment can be considered a promising strategy to reduce the environmental relevance of concentrated OMP mixtures in membrane retentates. The expected transformation pathways are consistent with the structure-dependent kinetic trends observed in this study. For phenolic compounds, including 4-OP, DES, E1, and E2, oxidation of the phenolic group, formation of hydroxylated and quinone-like intermediates, and subsequent ring opening may reduce the structural features associated with endocrine activity [32,33,53,54,58]. BPh and 4MBC are expected to undergo aromatic hydroxylation and oxidation of chromophoric structures. In contrast, CBZ and DEET may form hydroxylated, epoxidized, dealkylated, or ring-opened products through slower multi-step reactions [59,60]. The available literature generally indicates that activated PAA systems can reduce not only parent micropollutant concentrations but also their predicted toxicological relevance. However, the effect depends on compound structure and treatment conditions [26,58,61,62,63]. Thus, the results of the present study, together with the literature, support UV/PAA as an efficient and environmentally favorable retentate-treatment process. At the same time, the complete safety of the treated matrix should be confirmed in future studies by LC-HRMS and/or GC-HRMS suspect and non-target screening, residual PAA/H_2_O_2_ determination, TOC/DOC monitoring, and effect-based bioassays, particularly for aquatic toxicity and endocrine activity [58,59,60,61,62,63].

Recent studies confirm that the degradation efficiency of OMPs in UV/PAA systems depends not only on the contaminants’ chemical structures but also on operational parameters, including oxidant concentration, UV irradiation intensity, and water matrix composition [26,27,28]. These factors influence radical generation and reaction pathways, ultimately affecting oxidation efficiency.

There is relatively little literature on combining AOPs with membrane processes. Previous research has shown that AOPs such as UV/H_2_O_2_, photo-Fenton, and persulfate-based oxidation can effectively degrade OMPs in membrane retentates [20,25]. More recently, PAA-based oxidation systems have attracted increasing attention due to their high oxidation potential and lower risk of secondary pollution compared with metal-based catalytic systems [27]. The present study confirms that the UV/PAA system rapidly degrades a wide range of OMPs in nanofiltration concentrates. These findings support the concept of integrating nanofiltration with UV/PAA oxidation as an effective treatment strategy for surface waters contaminated with OMPs. Such an integrated approach allows both efficient removal of contaminants from the permeate stream and safe treatment of the retentate generated during membrane filtration. From a technological perspective, further research should focus on optimizing operational parameters, including oxidant dosage, reaction time, and UV intensity, as well as evaluating potential transformation products formed during oxidation. Nevertheless, integrating membrane filtration with UV/PAA oxidation represents a promising, environmentally sustainable approach for removing OMPs from water.

## 4. Conclusions

This study demonstrates that the combination of nanofiltration and UV/PAA oxidation is an effective strategy for removing OMPs from surface water and treating membrane concentrates generated during filtration. The results confirm that nanofiltration efficiently separates most investigated OMPs from the aqueous phase, although the degree of removal strongly depends on the physicochemical properties of individual compounds. Oxidation experiments on nanofiltration retentate revealed that UV/PAA rapidly degrades a wide range of pollutants in complex water matrices. The kinetic analysis indicated that the degradation rates varied significantly among compounds, reflecting differences in their molecular structure and susceptibility to radical oxidation. Compounds containing phenolic functional groups exhibited the highest reaction rates, confirming the dominant role of hydroxyl radical reactions in the degradation mechanism. The results also highlight the importance of oxidant dosage in determining treatment efficiency. Increasing the PAA concentration significantly enhanced degradation rates and enabled nearly complete removal of even relatively persistent compounds such as DEET and 4MBC. These findings suggest that proper optimization of oxidant dose and reaction time is essential for achieving effective treatment of membrane concentrates.

From a technological perspective, the results presented support the integration of membrane separation with advanced oxidation processes as a promising approach for addressing the challenge of OMP removal from water. Such hybrid treatment systems allow efficient water purification while also providing a feasible solution for managing retentate streams containing concentrated contaminants. Future research should focus on quantitative identification of reactive species contributions, evaluation of transformation products and residual toxicity after UV/PAA oxidation, and assessment of the energy efficiency and scalability of the process under real operational conditions.

## Figures and Tables

**Table 1:** The chemical composition of the studied surface water.

Chemical Parameter	Unit	Parameter Value
Color	mg Pt/L	74
Turbidity	NTU	4.4
pH	-	7.1
Conductivity	µS/cm	503
Oxidizability	mg O_2_/L	8.5
Ammonium Ion	mg/L	0.51
Nitrates	mg/L	<5.0
Nitrites	mg/L	<0.050
Total iron	mg/L	1.951
Manganese	mg/L	0.149
Total Organic Carbon	mg/L	40.7

**Table 2:** Selected physicochemical parameters of tested OMPs.

Analyzed Compound	Abbreviation	Structural Formula	Molar Mass, g/mol	Solubility in Water, mg/L	pK_a_	log K_OW_
N,N-diethyl-m-toluamide	DEET	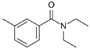	191.27	0.0196	<0	2.02
Benzophenone	BPh	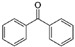	182.22	137	<0	3.18
4-octylphenol	4-OP	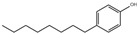	206.32	-	10.15	5.63
3-(4-methylbenzylidene)camphor	4MBC	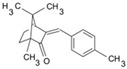	254.4	-	<0	5.14
Triclosan	TRC	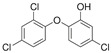	289.5	10	7.9	4.76
Carbamazepine	CBZ	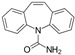	236.27	-	13.9	2.45
Diethylstilbestrol	DES	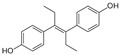	268.3	12	<0	5.07
Estrone	E1	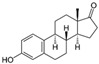	270.4	30	10.91	3.13
Estradiol	E2	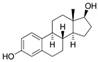	272.4	3.90	10.46	4.01
Clotrimazole	CTM	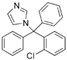	344.8	0.49	4.1	6.1

**Table 3:** Nanofiltration removal effect and OMP concentration in retentates.

OMPs	Retention Ratio [%]	Retentate Concentration [µg/L]
DEET	62.3%	591.26
BPh	94.7%	227.05
4-OP	21.2%	0.99
4MBC	98.4%	142.58
TRC	99.0%	42.21
CBZ	83.9%	8414.66
DES	97.5%	8.91
E1	96.9%	381.48
E2	98.2%	321.94
CTM	99.5%	219.27

**Table 4:** Kinetic parameters of OMPs degradation in the UV/PAA system.

Compound	k (min^−1^)	t_1_/_2_ (min)
4OP	1.05	0.66
BPh	0.98	0.71
E1	0.74	0.94
E2	0.63	1.10
DES	0.58	1.19
TRC	0.37	1.87
CTM	0.36	1.90
4MBC	0.14	5.14
CBZ	0.12	5.88
DEET	0.06	12.2

**Table 5:** The literature pseudo-first-order rate constants for the degradation of selected.

OMPs	Oxidation System	k (min^−1^)	Reference
BPh	PAA/Fe + UV	0.65	[33]
4OP	PAA/Fe + UV	0.73	[33]
4MBC	PAA/Fe + UV	0.12	[33]
DES	PAA/Fe + UV	0.52	[33]
E1	PAA/Fe + UV	0.68	[33]
CBZ	UV/H_2_O_2_	0.12	[52]
CBZ	photo-Fenton	0.10	[50]
TRC	photo-Fenton	0.33	[50]
TRC	UV/H_2_O_2_	0.41	[52]
E2	UV/H_2_O_2_	0.72	[52]
DEET	UV/H_2_O_2_	0.06	[51]

## Data Availability

The original contributions presented in this study are included in the article. Further inquiries can be directed to the corresponding author.

## Abbreviations

The following abbreviations are used in this manuscript:

4MBC	3-(4-methylbenzylidene)camphor
4-OP	4-octylphenol
BPh	Benzophenone
CBZ	Carbamazepine
CTM	Clotrimazole
DEET	N,N-diethyl-m-toluamide
DES	Diethylstilbestrol
E1	Estrone
E2	Estradiol
EDCs	Endocrine disrupting chemicals
GC–MS	Gas chromatography–mass spectrometry
NF	Nanofiltration
OMPs	Organic Micropollutants
PAA	Peracetic acid
TIC	Total inorganic carbon
TOC	Total organic carbon
TRC	Triclosan
USAME	Ultrasonic-assisted microextraction
UV	Ultraviolet radiation
